# Blockchain–Cloud Integration: A Survey

**DOI:** 10.3390/s22145238

**Published:** 2022-07-13

**Authors:** Abhirup Khanna, Anushree Sah, Vadim Bolshev, Alessandro Burgio, Vladimir Panchenko, Marek Jasiński

**Affiliations:** 1 Department of Systemics, School of Computer Science, University of Petroleum and Energy Studies, Dehradun 248007, India; asah@ddn.upes.ac.in; 2 Laboratory of Power Supply and Heat Supply, Federal Scientific Agroengineering Center VIM, 109428 Moscow, Russia; vadimbolshev@gmail.com; 3 Laboratory of Intelligent Agricultural Machines and Complexes, Don State Technical University, 344000 Rostov-on-Don, Russia; 4 Independent Researcher, 87036 Rende, Italy; alessandro.burgio.phd@gmail.com; 5 Department of Theoretical and Applied Mechanics, Russian University of Transport, 127994 Moscow, Russia; pancheska@mail.ru; 6 WWSIS “Horyzont”, 54-239 Wrocław, Poland; jasinski.lubin@gmail.com

**Keywords:** blockchain, cloud computing, decentralization, Blockchain-as-a-Service

## Abstract

Over the last couple of years, Blockchain technology has emerged as a game-changer for various industry domains, ranging from FinTech and the supply chain to healthcare and education, thereby enabling them to meet the competitive market demands and end-user requirements. Blockchain technology gained its popularity after the massive success of Bitcoin, of which it constitutes the backbone technology. While blockchain is still emerging and finding its foothold across domains, Cloud computing is comparatively well defined and established. Organizations such as Amazon, IBM, Google, and Microsoft have extensively invested in Cloud and continue to provide a plethora of related services to a wide range of customers. The pay-per-use policy and easy access to resources are some of the biggest advantages of Cloud, but it continues to face challenges like data security, compliance, interoperability, and data management. In this article, we present the advantages of integrating Cloud and blockchain technology along with applications of Blockchain-as-a-Service. The article presents itself with a detailed survey illustrating recent works combining the amalgamation of both technologies. The survey also talks about blockchain–cloud services being offered by existing Cloud Service providers.

## 1. Introduction

Blockchain is a new and emergent technology that is expected to change the way current markets work. It is a distributed digital ledger and is decentralized. With the current working capacity of blockchain, it has the potential to be the operating system of smart cities. Blockchain is technology that is open source and distributed and is used to record transactions between parties. It provides a way to develop a system that is both verifiable and secured. Blockchain is open source, so different versions of blockchain are available on the market. Each version is developed depending upon the different needs of the various industries. Blockchain is neither owned nor singly controlled by any one authority [1]. Blockchain technology is evolving at a swift pace. It started with Bitcoin, and now there are many types of blockchain. Organizations are developing different versions of blockchain depending upon their need and benefits. The critical development in blockchain innovation is that it permits its member to transfer resources across the Internet without the requirement of an incorporated outsider. The blockchain concept was created as the fundamental innovation behind the cryptocurrency called Bitcoin. Blockchain technology is currently being tested in many different asset management and procurement services for opportunities and has already led to many applications. Similar to today’s sophisticated flow of goods, there is a lack of transparency and trust. There are many intermediate people associated with high documentation requirements, which leads to time-consuming processes. Still, with a distributed blockchain system, the different interests of participants in a feed chain can be linked to a public register. Blockchain eliminates disclosure and accountability issues. Blockchain technology demonstrates high flexibility and has led to lasting changes in business models that could prove to be more profitable than existing ones [2]. Blockchain is an innovation that permits deals among numerous parties to be addressed in a dependable, non-impermanent, and appropriately secure way. A blockchain fills in as a grown-up trail to check the provenance in the food inventory network. In this way, it will prevent lawsuits. In international supply chains, blockchain will help if all the nations have a completely working general set of laws and execute the laws. The following are some of the prominent contributions of our work:

- Providing a systematic review of Blockchain technology with respect to Cloud Computing
- Exemplifying prominent works discussing the application of Blockchain–Cloud integration
- Providing a detailed bibliometric analysis across five real-world application areas of Blockchain–Cloud integration along with a reference architecture
- Exploring three key areas of Cloud computing which have had the maximum impact of Blockchain integration followed by a bibliometric analysis
- Identifying the top three allied technologies which complement Blockchain–Cloud integration for creating new-age solutions followed by their bibliometric analysis
- Providing a structured overview and description of publication patterns for Blockchain-as-a-Service and leading Cloud Service Providers rendering Blockchain integrated Cloud services.

The rest of the paper is organized as follows. Section 2 talks about Cloud computing along with its deployment models. In Section 3, we discuss benefits of Blockchain–Cloud integration followed by illustrating the key areas of Cloud computing wherein Blockchain can have a significant impact in Section 4. Section 5 discusses the notable works concerning application areas of Blockchain–Cloud integration. Section 6 talks about the adoption challenges pertaining to Blockchain–Cloud integration. In Section 7, we identify and discuss prominent allied technologies that are being combined with Blockchain–Cloud integration for creating new-age solutions across different research verticals. In Section 8, we describe our research methodology and present a detailed bibliometric analysis based upon existing academic literature. Section 9 discusses prominent Cloud Service Providers rendering services involving the integration of Blockchain and Cloud computing. Finally, Section 10 summarizes our findings and concludes the work.

## 2. Cloud Computing and Deployment Models

Cloud computing is an incredible model that enables clients and associations to purchase the administrations they need as indicated by their requirements. This model offers many types of assistance, such as stocking, arrangement, and helpful admittance to web administrations. Load adjusting is a typical issue in the cloud, making it hard to keep up with the exhibition of utilizations connecting Quality of Service (QoS) estimation and to meet the service level agreement (SLA). Cloud computing is an inventive organization-based framework that can deal with various solicitations from the cloud and offer quick support to clients. It is a computation and preparation model utilized in various parts of the world. Distributed computing can be utilized to further develop the estimating cycle utilizing high processing. It provides helpful, on-request admittance to tremendous processing assets such as CPU, memory, organization, worker, stockpiling, and applications. Moreover, these assets are frequently allotted to clients with the lowest assistance charge [3]. Cloud Computing is a new trend in information technology that is gaining a lot of attention from researchers. It provides an accessible, flexible, and scalable computing system over the internet for users. It enables them to use these resources remotely over the internet. Using these services, a lot of money is saved that was earlier spent to establish computing infrastructure and maintain it. We are living in a world where, every second, million and billions of data are processed and refined so as to provide a quality service. User bases in different firms share their data and activities to use the service and those data are processed so as to improve the services. As we are moving forward, this also is a threat to the privacy of user data. Accessing data from local machines was good in previous times, but these days the user has the power to sync their data to the cloud so that they can use services remotely from anywhere around the world. This opens another pipeline for direct targets to user data. So, it is important for firms to adapt a proper user-access control-management system. This article revolves around different algorithms and techniques to improve security and precision when accessing the services in cloud.

The characterization of cloud deployment models is performed, keeping in mind where the deployment of the model resides and who commands the network. Each model has a variety of necessities, and picking a model to fulfill the requirement of the client or organization is significant. One of the main deployment choices that you will have to make is which model to choose as per your requirements and needs. Every cloud-deployment model offers different features or services and has a different variety of alternatives depending upon its costs. Therefore, to select the correct model for your organization, you should make an intelligent choice. The last decade has seen significantly more organizations become dependent on Cloud computing for better effectiveness and adaptability, as well as faster time to take products to the market. However, the response as to which cloud model is the best fit for an organization relies upon the organization’s demands and needs. It assists them with accomplishing the long-term advanced objectives as a feature of their system. Picking the right one from the different kinds of cloud deployment models is fundamental. It would guarantee your business is provided the protection, security, adaptability, consistence, compliance, and cost-adequacy it needs. Different types of cloud deployment models are listed below.

### 2.1. Private Cloud

Private cloud, as its name suggests, is the cloud that is maintained, controlled, and managed by an organization. Usually, the entire infrastructure is in the datacenter that an organization controls or manages. In this way, the organization is liable for procurement, upkeep, and support services. Resources in the private cloud are confined within a single organization. A private cloud is a committed environment for one (client). You do not impart the infrastructure to some other clients. When we need our information to be secure and we want assets that ought to be restricted to a specific measure of groups, private clouds are preferred. Private clouds are utilized by a restricted measure of individuals, e.g., where an association sets up its cloud only for the utilization of its workers. Only the individuals working in that association can access it. The information put away on the cloud is available just to the chosen group of individuals with the components of improved security, quality, and protection. Generally, all the equipment is yours. A few examples of private clouds are IBM Bluemix, Rackspace, Red Hat OpenStack, VMware, and Microsoft Azure Stack. The private cloud is beneficial for putting away corporate information where just an approved work force can gain access to resources, i.e., data privacy is important in this type of cloud. Private clouds are also great for security purposes. There are more significant levels of safety and better access, as resources are distributed inside the same organization. Private clouds support legacy systems [4].

### 2.2. Public Cloud

The public cloud is accessible for everybody, for example, every last one of us is qualified for use and can store information. Generally, single clients utilize it. Mid-level organizations’ cloud service providers offer their resources and services to everybody as per their demands. The public cloud is facilitated on the premises of the service provider. Being available to everyone, it does not ensure security by and large. Thus, it is appropriate for the organizations for which security is definitely not a main pressing issue while managing their information. Examples of public clouds are Google App Engine, Salesforce Heroku, etc. Public cloud models are ideal for associations with developing and fluctuating requests. Accordingly, you pay a cloud service provider for an infrastructure, computation, and networking service. Moreover, the public cloud is utilized when investment is minimal, i.e., there is no enormous forthright expense and is great for organizations where they need quick access to resources. This model also fit best where the whole infrastructure is stored with the cloud provider only. Moreover, there is no need for management of the infrastructure.

### 2.3. Hybrid Cloud

A hybrid cloud is a blend of both public cloud and private cloud where a client can utilize benefits of both parts. Assuming you need for a few pieces of your information not to be open to everybody, you can store it to the private part of the hybrid cloud, and, for the other non-confidential and public type resources and information, you can use public part of the cloud. It provides the infrastructure at a sensible cost which is higher than the public cloud, but also less expensive than private cloud. It is restricted to the organizations that can isolate their information into private and public crucial parts. Many organizations have many resources of their own, so they would like to utilize few resources of their own and want to borrow a few from cloud vendors. In such scenarios, this model plays a vital role and is a good choice. Thus, this model is the second-most famous model. This model allows organizations to use some of the on-premises available framework and the rest of the resources of the public cloud. This is one of the very intelligent choices, and it is valuable too. Using only public cloud causes security or information-breach issues and, for a few organizations, security is one of the critical requisites, so they utilize the hybrid cloud with advantages of using public cloud. The model is costly and expensive to use and execute.

### 2.4. Community Cloud

The community cloud is used for a gathering of individuals that have comparative interests, regularly known as a community. At the point when at least two associations have comparative necessities, they counsel to a community cloud that holds services that are normal for organizations. It can end up being of extraordinary advantage to the organizations that are dealing with joint tasks/projects. Data transmission or limits on the capacity are fixed in this type of deployment model. This cloud is committed to a couple of organizations from similar communities. A community cloud is neither public nor private, as it is not open for the public, and it is also not governed by a single organization or vendor but is governed by group of organizations. A community cloud is shared by a gathering of associations that have a common reason or objective. The cloud is mostly used to assist them with accomplishing that objective. A community cloud is ideal where investment is smaller and setup benefits are good.

### 2.5. Multi-Cloud

In a Multi-Cloud model, each cloud vendor is used in turn. This model uses private and public clouds, and is very much similar to a Hybrid cloud. You would utilize more than one type of cloud in this model. The Multi-cloud model gives you far better accessibility of services than any other type of deployment model. One more justification behind utilizing a multi-cloud is the point at which you want a particular infrastructure or services from one public cloud and explicit help from another public cloud. The Multi-cloud model provides various options to the organizations for expanding service dependability.

## 3. Benefits of Blockchain–Cloud Integration

Blockchain innovation is a vital innovation in the world that will allure businesses and other sector areas to utilize that innovation to work on various services. Blockchain is a progressive innovation that may innovate current market trading exchanges. The forthcoming Industry 4.0 interfaces the most recent advancements, for example, blockchain, AI, distributed computing, and IoT for improving the usefulness and productivity of their frameworks [5,6,7,8]. There are several benefits of blockchain and cloud integration; here, we are discussing the popular advantages of blockchain and cloud integration.

### 3.1. Decentralization

In cloud computing, information is kept in a centralized server, which is one of the major issues from the perspective of security; this can be overcome by accepting blockchain in cloud computing. In IoT and cloud computing, a significant issue is a reliance on a brought-together server to oversee information and for making choices. The blockchain can give an answer to these issue as in the decentralized framework different duplicates of similar information are put away on numerous hubs which eliminates the chance of failure of the whole system. Additionally, the deficiency of information cannot be an issue, as numerous duplicates of the information are available on different hubs. Blockchain with integration with cloud computing is a possible good solution for decentralization and could provide total privacy to users.

### 3.2. Data Security

Blockchain systems by default inherit data security features. Loads of data are transacted and loaded into the cloud, so security of data is one of the main concerns in cloud computing that is provided by blockchain–cloud integration in different sectors. Even the storage of information on the cloud in the field of the Internet of Things (IoT) is a major test. IoT devices put away information, such as the individual data of the house proprietor including their voice accounts, video films, their family things, their property, and their own propensities in cloud, and the destruction of this information can hurt the individual security including assaults, theft, and illicit selling of the individual’s information for cash. These conditions represent a danger to the IoT and cloud foundation. The answer to this issue is the utilization of blockchain in cloud computing, which has the capability of providing upgraded security to the entire engineering.

### 3.3. Adaptability

In blockchain applications, the quantity of exchanges in blockchain organizations can be tremendous. Blockchain has amazing information handling techniques to have large-scale exchanges for empowering adaptable blockchain services. Therefore, in this regard, cloud computing can provide on-demand services for blockchain activities, because of its scalability abilities. In this way, the blend of blockchain and cloud computing can provide an exceptionally versatile coordinated system.

### 3.4. More Efficient Supply Chain Management

Blockchain is the technology that will help to develop cost effective and more efficient ways of supply chain management. Blockchain enables better end-to-end tracking of goods and services, and it can be incorporated into cloud computing for much better results for the supply chain industry. A significant challenge of the supply chain management industry is to consistently monitor all vehicles in its organization, their present areas, time for which a vehicle remained in one spot, to set up correspondence between different vehicles. In a similar way, tracing different services such as products, parcels, etc., deal with issues because of the centralized methodology of their design. Blockchain has sufficient potential for tracing these goods and services.

### 3.5. Tolerance of Faults and Errors

Blockchain requires replicating information over the network over different servers, and this can be achieved by using Cloud. This will also limit the single point of risks from the point of view of the disturbance of any cloud hub, so that blockchain can provide uninterrupted services.

## 4. Impact Areas of Blockchain in Cloud Computing

Cloud computing is one of the key complementing technologies that support the creation and functioning of blockchains. Some of the prominent advantages of Blockchain–Cloud integration have been discussed in the previous section. In this section, we explore the key areas of Cloud computing which have undergone significant transformations as a result of integration with Blockchain technology. “Security”, “Privacy”, and “Storage” are areas of Cloud computing wherein numerous advancements have been observed since its integration with Cloud. Section 6 discusses the bibliometric analysis for all three areas and demonstrates the interests of researchers through publication patterns. Moreover, it has been realized that, to facilitate the growth of Cloud computing, it is essential to overcome the roadblocks of data security and privacy for which blockchain seems a perfect match. Table 1 summarizes some of the prominent works for the mentioned areas.

Data security and user privacy are major concerns for Cloud adoption. Blockchain integration with Cloud has the potential to mitigate the challenges of security and privacy [10]. The distribution of vast volumes of data over a Blockchain–Cloud environment improves accuracy and minimizes cost [22]. Furthermore, improved access control mechanisms can be implemented in a Cloud environment through means of blockchain integration. As most Cloud organizations follow a centralized access control mechanism, integration with blockchain will infuse decentralization, preventing tampering or leakage of data via internal cloud managers [15]. Blockchain-enabled cloud solutions will ensure an efficient framework for identity access control, thereby supporting privacy protection [25,26,27,28]. Cloud auditing is another such area wherein privacy is of the utmost importance, as it involves the tracking and logging of all operations and their relevant data. Blockchain integration with Cloud will ensure preserving the provenance of data from being violated within the cloud ecosystem. The decentralized nature of blockchain will facilitate securing the origin of data and information on data owners, thereby solving one of the major concerns of cloud storage applications.

Blockchain-data-based cloud data entry protection mechanism. Over the years, cloud computing developed a lot, but data security and trusted computing remains a challenge in many cloud applications. Though scholars have conducted many kinds of research, and many models have been proposed by them, including the data integrity test and multiparity calculation; they still face problems such as excessive computational complexity and the lack of scalability. Blockchain technology has emerged as a new dynamic computing paradigm in which data blocks present in the database are generated through a crypto-graphic algorithm. The key features of blockchain, such as decentralization, anonymity, auditability, and persistence have made it possible to use blockchain technology in many fields. The topic discussed in this paper is how Blockchain technology can be applied to cloud computing using the security mechanism to improve the performance in secure storage and secure computing. The paper analyzes the requirements of security in cloud storage data and also examines cypher text access control technology and integrity verification technology.

Ref. [29] discusses the distributed virtual machine agent model deployed in the cloud using mobile agent technology. The multi-tenants cooperate with each other ensuring data trust verification through the virtual machine agent. The virtual machine agent can complete the monitoring and verification tasks, which are essential for building the blockchain-based integrity protection mechanism. This integrity-protection mechanism based on blockchain is built utilizing the virtual machine proxy model and the unique hash value generated by the Merkel hash tree. It is used in monitoring the data change utilizing the smart contract in the blockchain database and the data that is used in the current time. Here, users can issue a message for the data tempering and the creation of blockchain-based cloud data integrity verification scheme is completed by “block-and-response” mode [30]. Ref. [31] examines the decentralized virtual machine specialist model deployed in the cloud utilizing the mobile agent innovation. The multi-tenants cooperate with one another, guaranteeing the information trust check through the virtual machine specialist. The virtual machine specialist can observe and check jobs, which is essential for building the blockchain-based trustworthiness insurance instrument. Here, users of the system can signal information tampering and the construction of a blockchain-based cloud information-verification system [32]. Ref. [33] constructs a technology application scheme of blockchain-based cloud computing by combining the plus points of blockchain and cloud computing. This scheme provides the protection and integrity check of the data. Moreover, the multi-parity scheme that is based on the blockchain has been projected. The security mechanisms and algorithms in blockchain and the general schemes of scalable multiparity computing have been discussed and studied.

## 5. Application Areas of Blockchain–Cloud Integration

**Figure 1:**
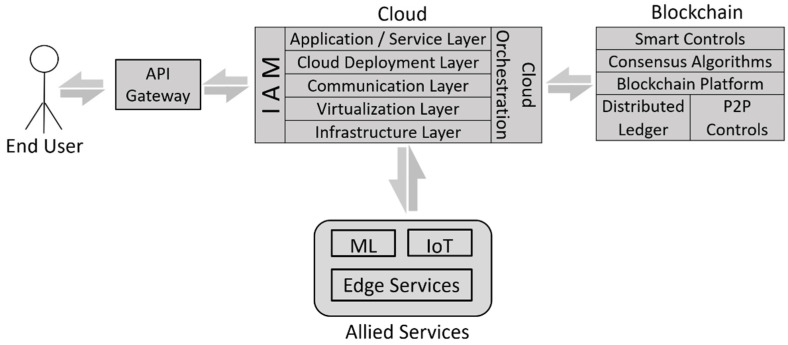
Blockchain–Cloud Reference Architecture.

The concept of Cloud computing has had a prevalent computing model for almost a decade. The last couple of years have seen a surge in the number of organizations migrating their businesses to the Cloud. The ubiquitous availability of resources, attractive pricing models, customized solutions, and numerous market players are some of the factors that contribute to the success of Cloud computing. In recent years, Cloud has emerged as a synonym for computing and storage capabilities that can be accessed over the internet. Cloud has transformed itself into a generic computing paradigm that supports and compliments the functions of other new-age technologies. Containerization, AI, Internet of Things, and Big data analytics are some of the services to have become a part of the larger pool of services being offered by leading Cloud Service Providers (CSPs). Researchers are truly of the belief that the integration of Cloud with other technologies can result in the creation of more robust, scalable, and secure applications. Blockchain technology is the newest entrant to the list of technologies looking forward to Cloud integration. Blockchain has merged as a new-age technology and has been exciting researchers and industry professionals for the last couple of years. The distributed ledger technology allows individuals to conduct transactions in a secure and automated manner. Figure 1 represents a reference architecture for Blockchain–Cloud integration. In this section, we discuss the integration of Blockchain technology with Cloud computing. During the course of our research, we carried out a comprehensive literature review and identified five key application areas of Blockchain–Cloud integration: (1) Healthcare, (2) Supply Chain, (3) Finance, (4) Smart Cities, and (5) Agriculture. Table 2 summarizes some of the prominent works for the above application areas.

### 5.1. Smart City

In the recent past, the concept of “smart cities” has attracted considerable curiosity among researchers across the world. The rise of Cloud computing and the Internet of Things has provided immense infrastructure support for the creation of smart cities. Blockchain being the new member of the bandwagon of technologies, aims to facilitate the creation of citizen-centric applications for a smart city environment. Blockchain has the ability to operate an entire smart city in an autonomous fashion when combined with IoT, AI, and Cloud computing. Blockchain-enabled IoT solutions are becoming more and more popular among industry players as they support ubiquitous sensing capabilities and intelligent information communication and processing. Blockchain enables the trusted and transparent exchange of information between IoT devices through the use of smart contracts and consensus algorithms. Energy trading and distribution platforms, traffic management systems, smart homes, and IoT applications are some of the many application areas of blockchain integration in smart cities. Moreover, blockchain has the potential to enhance the extent of e-governance, thereby improving citizen participation and formulation of government schemes in a smart city.

### 5.2. Smart Healthcare

Blockchain has revolutionized the healthcare sector by providing applications for health record management, medical insurance claims, and pharmaceutical supply chains. The technology has enabled health professionals to manage patient data in a secure manner without third-party intervention. Blockchain has enabled government authorities to roll out better healthcare schemes on the basis of the health records of its citizens. Diagnostic reports of patients can now easily be communicated to doctors and insurance firms for the purpose of faster claim settlements. The immutable nature of blockchain allows it to ensure trust and accountability and facilitate the creation of a patient-centric healthcare system. Complementary technologies of the likes of AI, Cloud, and IoT are a great success when combined with blockchain in creating modern day healthcare systems. The Internet of Medical Things, Edge-based healthcare systems, and AI-enabled medical imaging systems are some of the many areas of blockchain integration. Post pandemic, numerous works have been published discussing the applicability of Blockchain technology, IoT, and AI for the purpose of contact tracing and vaccination certificate distribution and validation.

### 5.3. Supply Chain

The recent studies in the area of blockchain technology depict its popularity beyond the realm of cryptocurrencies. Supply Chain Management (SCM) is one of the many leading areas discussing the applicability of blockchain technology. Numerous works in the past have been published stating the use of blockchain technology for managing food, agriculture, retail, hospitality, and pharmaceutical supply chains. SCM has always been a challenging task for organizations, and, specifically post pandemic, the complexity and challenges have increased manyfold. SCM holds the key to numerous economic activities of a country, and any disruption may lead to large fiscal deficits and job losses. Companies need to modernize SCM practices in order to stay relevant and possess a competitive advantage. Blockchain integration with SCM serves this purpose by providing viable methods of asset tracking while ensuring security and data integrity. Data being generated at every stage of the supply chain are recorded in the form of transactions. Blockchain-enabled systems are transparent in nature and support the real-time data collection of a product across the entire supply chain. The entire lifecycle of a product can be managed using blockchain technology while ensuring quality control. Blockchain technology has the potential to contribute to various aspects of SCM such as physical and digital asset tracking; tracking orders and payments; and managing invoices, licenses, and copyrights. The decentralized nature of blockchain enables a continuous information flow and facilitates the seamless sharing of this information between suppliers, vendors, manufactures, and end-user customers across the entire supply chain. The absence of a central authority, the presence of a distributed ledger, and a trust-based ecosystems enable blockchain to weave a network of complex assembly lines.

### 5.4. Agriculture

Blockchain technology can be seen as an enabler for the agriculture sector. Removal of numerous intermediatory entities and direct communication between farmer and the end user are the biggest advantages that blockchain technology provides to the agriculture sector. Smart-contract-enabled trading platforms are enabling farmers to sell their produce at favorable rates directly to the end customers. Blockchain is a great supporter of the concept of information-intensive farming that involves Agri data assimilation and intelligent decision making. Smart agriculture that involves the use of new-age technologies is inevitable for the farmers to adopt as it is the only means of rural development and revitalization of the farmer economy. A Blockchain-enabled token-based economy can provide a secure and efficient trading platforms for assisting farmers in trading their crop produce.

## 6. Blockchain–Cloud Adoption Challenges

### 6.1. Limited Interoperability

Multiple, enterprise-grade blockchain platforms exist that can handle enterprise transactions. However, there are no standards allowing them to communicate with each other. For widespread adoption to occur, there needs to be some kind of proven cross-platform system that allows businesses running on different blockchain platforms to collaborate. However, currently, no such system exists. It would be hard for a company using Hyperledger Fabric to coexist with its partner using Corda services without having compatibility issues. Blockchain Platform Services have begun to address this issue. Hyperledger and Enterprise Ethereum Alliance, two of the most popular enterprise blockchain platforms, have decided to work together to define standards to enable interoperability. However, there is still more work to be completed to address this concern and enable the mass adoption of enterprise blockchains.

### 6.2. Regulatory Issues

Enterprise blockchain technology is still in its infancy, with a small number of pilot projects being completed. This makes it difficult for lawmakers to set forth adequate rules and regulations for managing enterprise blockchain networks. For a vast majority of enterprises, these networks will be distributed across the world, making it a complex process for governments to establish jurisdiction. Globally dispersed blockchain networks will make it hard for governments to establish rules around data storage and sharing. Given the network’s complexity, once an illicit transaction has occurred, authorities may find it challenging to trace it down and identify the legal obligations of the parties involved.

### 6.3. Regulation Deficiency

One of the major challenges in the blockchain industry is that there is no regulation for blockchain in any organization. Most organizations in the IT world are opting to use blockchain technology to make transactions. Even sometimes, many products depend on this technology. Moreover, with no specific regulation, it is becoming difficult to manage and regularize, as no one is following any rules that are specific to the blockchain market. So, to make blockchain work and to overcome challenges in the application of blockchain in the real world, the government and other such governing parties need to set up rules and regulations or protocol for blockchain to be successfully adopted in the market.

### 6.4. Tensions Regarding Criminal Activities and Cybersecurity

News about people using digital currencies for criminal activities and conducting dubious transactions has painted a negative picture of blockchain technology. According to blockchain data company Chainalysis, just 0.34% of all cryptocurrency transactions in 2020 were illegal, but there was a 311% increase in ransomware incidents. The thought of such risks is holding some enterprises back from adopting blockchain technology. One of the most attractive features of blockchain technology is that it is secure by nature, yet companies are not comfortable with deploying blockchain in their operations. Blockchain is vulnerable to phishing scams, but this generally affects cryptocurrency transactions and not enterprise blockchains. Ransomware attacks still pose a threat to enterprise blockchains. However, strong multi-factor authentication can be used to stop these.

### 6.5. Uncertain ROI

The return on investment from a blockchain solution is a major concern for companies implementing blockchain technology. As discussed earlier, blockchain adoption is a cost-intensive affair, which makes ROI crucial. According to an IBM study, organizations expect just around 20% ROI on their blockchain investments in the next 4 to 5 years and about 50% ROI in 10 years. Predicting ROI from a blockchain project is a complex process, and there is no proven formula for the blockchain ROI calculation. This is a major challenge that holds organizations back from adopting blockchain technology.

### 6.6. Integration of Blockchain–Cloud with Legacy Systems

Integrating legacy systems with the new blockchain system is a major challenge the industry is facing nowadays. To completely integrate blockchain with legacy systems designers have to restructure the complete system and have to integrate different technologies. While integrating blockchain with legacy systems, a few issues arise such as there is a shortage of skilled labor (or you can say there is a lack of developers with the proper skill set for the blockchain industry). Due to this, sometimes organizations have to rely on a third party that further softens this problem. Therefore, to complete this transition, organizations have to invest a significant amount of their time and resources. Most companies nowadays are encouraging the transition to blockchain, as there are many indices of loss of data. Companies are not willing to make changes to their databases and this contributes to great risk. Data loss or data corruption are two major losses IT companies may face, and, with the adoption of blockchain technology, these companies benefit. With the growing market demands, new companies are investing and developing new systems or ways to integrate blockchain and legacy systems. One example of this type of product is the Modex Blockchain database. The product is developed with the mind that users who use this do not have much knowledge of blockchain, and thus it reduces the chances of the loss of sensitive data.

### 6.7. Low Scalability

Scalability came as another challenge in implementing blockchain. Blockchain works fine in the case of a small number of users, but what will happen when mass integration takes place. When it comes to the number of users, Ethereum and Bitcoin have the highest number of users on their networks, but they are having difficulty managing and dealing with them. With the increasing number of users on the network, the transition takes a longer process. Thus, the transition costs are higher than usual and, in turn, result in more users on the network. It can take days, with the entire process of transaction leading to making the technology less and less lucrative and worthwhile. A few other blockchain technologies adopted showed a faster output but eventually slowed down when the number of users logging into the system increased. Thus, this challenge needs to be managed quickly, as it is making the entire technology dull.

### 6.8. High-Energy Consumption

Another challenge that comes up is energy consumption. The majority of blockchain technology follows the blockchain framework and uses Proof of Work as a consensus algorithm. However, Proof of Work needs computational power to keep the system alive. Mining leads to solving complex equations using the computer. This, in turn, leads to the PC utilizing more and more electricity in overcoming the situation when you start mining. As of now, the miners are utilizing 0.2% of the total electricity, and if it continues to increase then they might take even more power than the world can provide. This becomes one of the primary challenges in adopting blockchain technology that needs to be dealt with. But how? Blockchain can use consensus methods to authorize transactions. Consensus algorithms used require much less energy to process. This is the only way to make blockchain technology a blessing again.

## 7. Allied Technologies

The combination of innovative technologies such as Cloud Computing, Blockchain, and IoT has proven to be beneficial for designing new-age IoT solutions. Even through IoT and Blockchain are distinctive technologies, they both have the potential to come along and create futuristic solutions. Blockchain can be seen as a solution to existing concerns of privacy and security in the IoT domain [66,67,68]. The use of blockchain technology along with cloud computing can be seen as a promising solution for numerous IoT applications. Identity management, data storage, and autonomous processing are some of the impact areas wherein Blockchain–Cloud integration can prove beneficial for IoT applications. Supply Chain Management, Smart cities, and Intelligent healthcare systems are some of the most popular research areas of IoT which have recently found the applicability of Blockchain–Cloud integration [69,70]. Smart contracts have proved their significant worth for managing and operating supply chains and smart cities. The concepts of the Internet of Vehicles (IoV) and Device-to-Device communication are majorly supported by Blockchain–Cloud integration, wherein blockchain supports the secure exchange of data between devices and establishes trust and traceability among various passengers. The availability, interoperability, and standardization of data is ensured by the Cloud end. Talking of intelligent healthcare systems, data collected from a variety of sensors is exchanged, stored, and processed using Blockchain–Cloud integration. The combination of these technologies assists the doctor to monitor the health of their patient on a real-time basis with utmost efficiency and privacy protection.

The rapid growth in the number of IoT devices has led to large volumes of data being transmitted to the Cloud end, thus resulting in massive levels of network bandwidth consumption. In order to resolve the problems of centralized failure and large bandwidth consumptions, an extension of Cloud was introduced, known as edge computing. Data collected by various IoT devices are stored on different edge servers for faster processing and high-frequency real-time access [71,72,73]. Storing and processing sensitive data over edge servers continues to be a challenge and thereby involves an integration with blockchain technology. Blockchain helps to create a secure decentralized system that supports privacy protection, encrypted data storage and access, secure access control, intrusion detection, and effective authentication mechanisms. The consensus mechanisms of blockchain assist in managing distributed databases spread across multiple edge servers. Blockchain–Cloud integration with edge computing helps to create a distributed edge-computing environment that supports the tracking of assets and integrity of transactions among IoT devices [74]. The decentralized nature of the system will help to prevent any malicious attacks from insiders, along with maintaining data transparency. Blockchain–Cloud integration will ensure enhanced cooperation between IoT devices and edge servers. Blockchain–Cloud integration with edge computing is mutually beneficial, as edge servers assist in managing mining time and improve the scalability of the blockchain. Blockchain coupled with 6G-enabled edge services and edge-computing-based autonomous vehicles are some of the most prominent research areas for researchers working on Blockchain–Edge integration.

The third biggest allied technology for Blockchain–Cloud integration is AI. The massive production of data from IoT devices, web applications, and social media websites has given rise to numerous applications of AI and deep learning [75,76,77,78]. The complexity of machine learning models and the large variety and volume of data which they process make it a mandate for them to be hosted on Cloud. However, the centralized nature of AI could result in issues relating to data breaches and the authenticity of the data [79]. The combination of blockchain and AI gives rise to a new concept called Decentralized AI, wherein data are stored and shared in a secure manner using digital signatures and encryption techniques are stored in a decentralized manner. Trusted decision-making and proper data governance mechanisms are in place one Blockchain–Cloud integration is coupled with AI. The use of smart contacts can be extremely beneficial for creating autonomous systems, as decisions taken by an intelligent machine can be verified and validated by miner nodes of the blockchain. Moreover, blockchain integration with AI can support the concept of decentralized learning and involving the secure and trustworthy distribution of decision outcomes, hyperparameter values, and weights in neural networks [80,81,82,83]. Decentralized learning will involve the coming together of autonomous intelligent machines that can contribute to accurate decisions. Blockchain–Cloud integration with AI will the support storing and processing of tamper-proof data that can be cryptographically signed before distribution and subsequently validated before further processing. Smart city is one such specific domain that finds the applicability of all allied technologies along with Blockchain–Cloud integration [84]. It is through the use of these allied technologies that we can create a truly smart, autonomous, and sustainable smart city. Table 3 presents a comparative analysis of some of the prominent works concerning Blockchain-Cloud integration with allied technologies.

## 8. Literature Survey

### 8.1. Methodology

This paper follows a multi-step research methodology for analyzing publishing patterns in the field of Blockchain technology and Cloud computing. A survey was conducted based on the Scopus database [100]. Articles published between 2017 and 2021 were considered for the survey. During the process of the publication search, we ensured that the papers selected were written in English language. The primary step involved searching publications on the basis of keywords such as “Blockchain AND Cloud”. We ensured that the keywords were mentioned in either the paper’s abstract or title. The results of the first step led to the identification of three prominent sub-domains wherein Blockchain and Cloud find applicability. The subsequent step involved searching publications on the basis of keywords “Blockchain AND Fintech”; “Blockchain AND Smart City”; “Blockchain AND Healthcare”. Furthermore, we analyzed our search results on the basis of three dimensions which led to enhanced insights with respect to the research being conducted in Blockchain technology and Cloud computing. Finally, we have a subsection discussing publications focusing on Blockchain-as-as-Service (BaaS). The keyword “BaaS” has been used for the classification of works that talk about either the implementation or the use of Blockchain-as-as-Service. The following is the set of keywords that were searched during our survey.

1. Blockchain + Cloud Computing
2. Blockchain + Cloud + Smart Healthcare
3. Blockchain + Cloud + Finance/DeFi
4. Blockchain + Cloud + Agriculture
5. Blockchain + Cloud + Supply Chain
6. Blockchain + Cloud + Smart City
7. Blockchain + Cloud Security
8. Blockchain + Cloud Privacy
9. Blockchain + Cloud Storage

### 8.2. Blockchain–Cloud Application Areas

**Figure 2:**
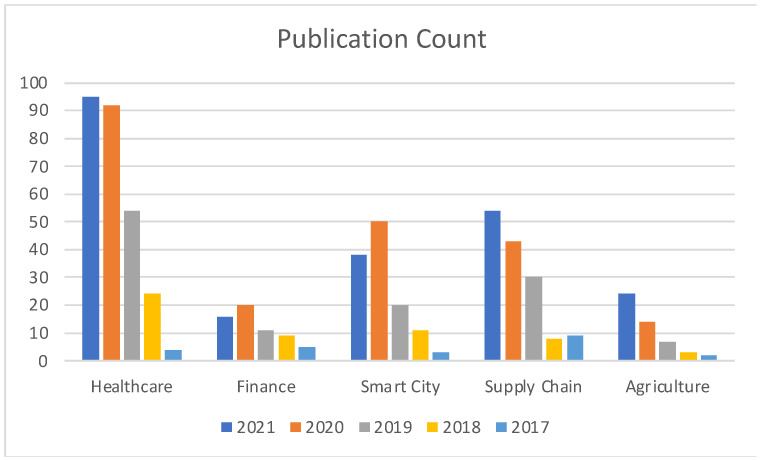
Publication count comparison among application areas.

Figure 2 illustrates the comparison between prominent application areas with respect to the number of publications from the year 2017. Our survey indicated the abovementioned five subdomains as the most notable ones with respect to works concerning Blockchain and Cloud integration.

**Figure 3:**
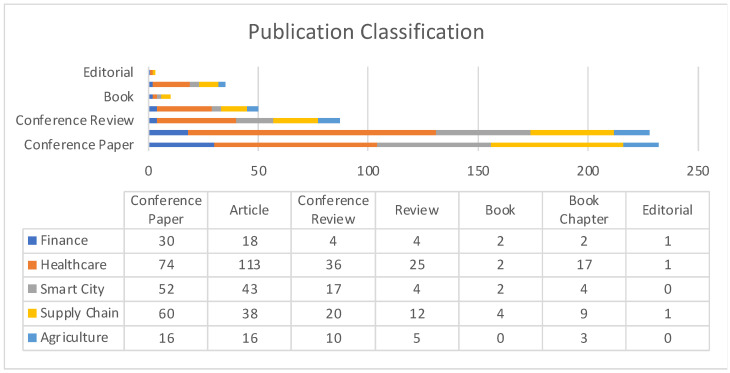
Publication classification comparison among application areas.

Figure 3 describes publication distribution in terms of article classification such as conference papers, book chapters, articles, conference review papers, etc. Except in the case of healthcare, all other sub-domains have conference papers constituting the largest section in terms of works being published.

**Figure 4:**
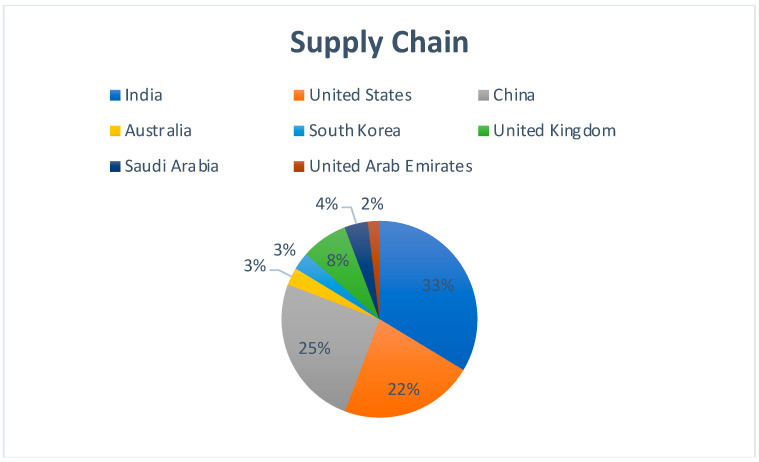
Region-specific publication distribution for Blockchain–Cloud and Supply Chain.

Figure 4 represents the country-wise publication distribution for works concerning the use of Blockchain and Cloud in the area of Supply Chain Management. India leads from the front, followed by China and The United States. India having the maximum numbers indicates the creation of modern supply chain solutions, which is an essential part of the larger plan to be the global manufacturing hub.

**Figure 5:**
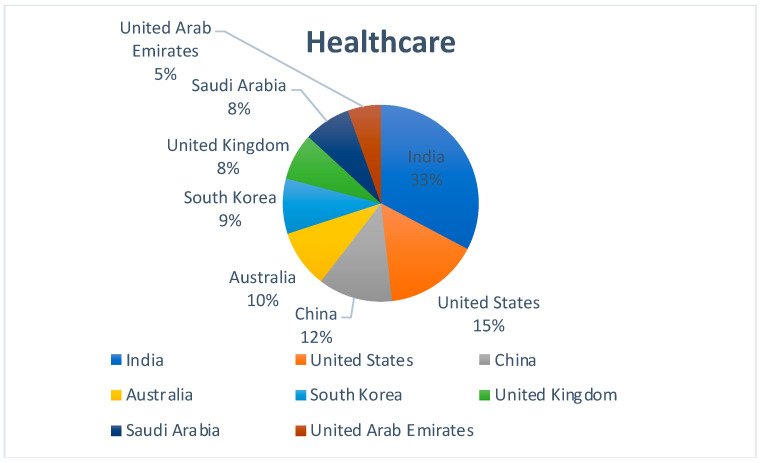
Region-specific publication distribution for Blockchain–Cloud and Healthcare.

Figure 5 represents the country-wise publication distribution for works concerning the use of Blockchain and Cloud in the healthcare sector. India is the largest contributor followed, by the Unites States and China. The graph reinstates the fact that countries such as India and China are aggressively working on creating new-age healthcare solutions that combine the use of multiple technologies.

**Figure 6:**
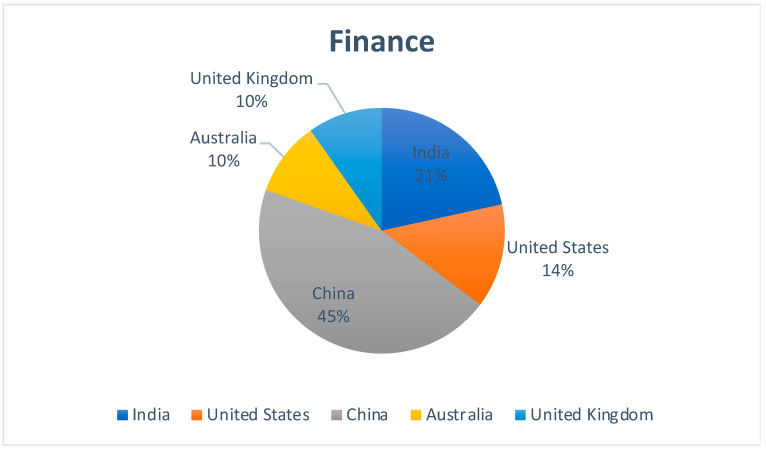
Region-specific publication distribution for Blockchain–Cloud and Finance.

Figure 6 represents the country-wise publication distribution for works concerning the use of Blockchain and Cloud in the area of Finance. The graph represents the top five regions for works involving the use of Blockchain–Cloud integration for Finance related applications. China has the highest numbers, with India and The United States following.

**Figure 7:**
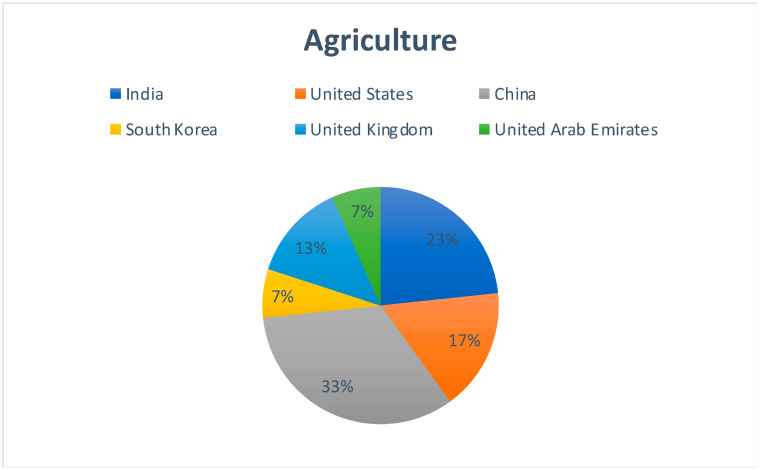
Region-specific publication distribution for Blockchain–Cloud and Agriculture.

Figure 7 represents the country-wise publication distribution for works concerning the use of Blockchain and Cloud in the area of agriculture. China, India, and the United States are the top runners. The large numbers coming from China indicate the efforts which they have been making towards sustainable agriculture.

**Figure 8:**
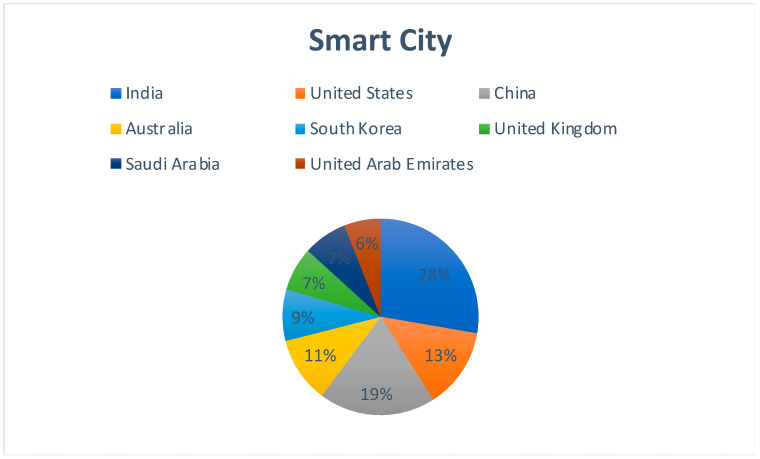
Region-specific publication distribution for Blockchain–Cloud and Smart Cities.

Figure 8 represents the country-wise publication distribution for works concerning the applicability of Blockchain–Cloud integration for the creation of smart cities. India and China are the most prominent sources of publications, which is a direct correlation to the fact that both countries have made significant investments in relation to creating smart cities in the past few years.

### 8.3. Cloud Impct Areas

**Figure 9:**
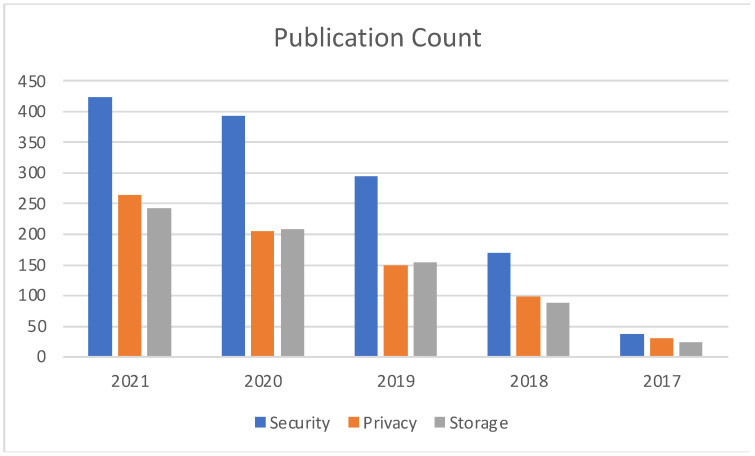
Year specific Publication Count comparison among Cloud impact areas.

Figure 9 illustrates the publication comparison between key areas of Cloud computing which have seen the highest impacts as a result of blockchain integration. Our survey indicates that security, privacy, and storage are the top three segments of the Cloud ecosystem, to which blockchain technology has significantly contributed.

**Figure 10:**
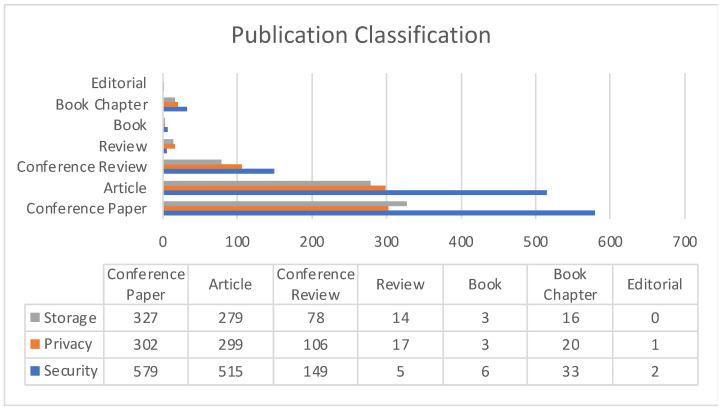
Publication classification comparison among Cloud impact areas.

Figure 10 represents the publication classifications of all three Cloud impact areas since the year 2017. The graph clearly indicates conference papers to be the largest section in term of works being published. Cloud Security is one particular impact area which has seen the highest number of publications in recent times.

**Figure 11:**
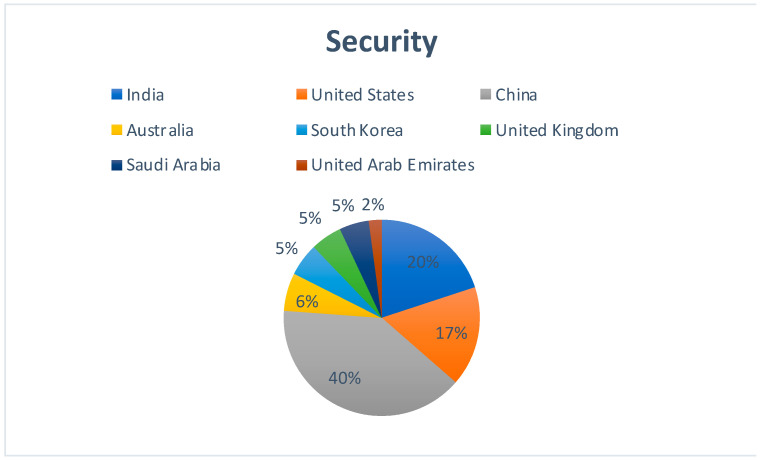
Region-specific publication distribution for the impact of Blockchain–Cloud integration on Cloud Security.

Figure 11 represents the country-wise publication distribution for works concerning the applicability of Blockchain technology and Cloud security. China has a significant lead on the rest of its contemporaries. Cloud security is one such impact area which is seeing equal numbers of contributions from countries such as South Korea, the United Kingdom, and Saudi Arabia.

**Figure 12:**
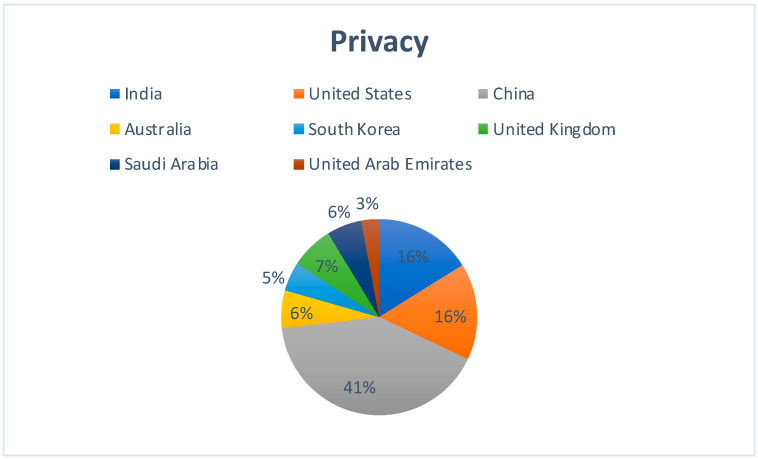
Region-specific publication distribution for the impact of Blockchain–Cloud integration on Privacy in Cloud.

Figure 12 represents the country-wise publication distribution for works concerning the applicability of Blockchain technology and privacy aspect of Cloud computing. China has a 41% share of the total number of publications.

**Figure 13:**
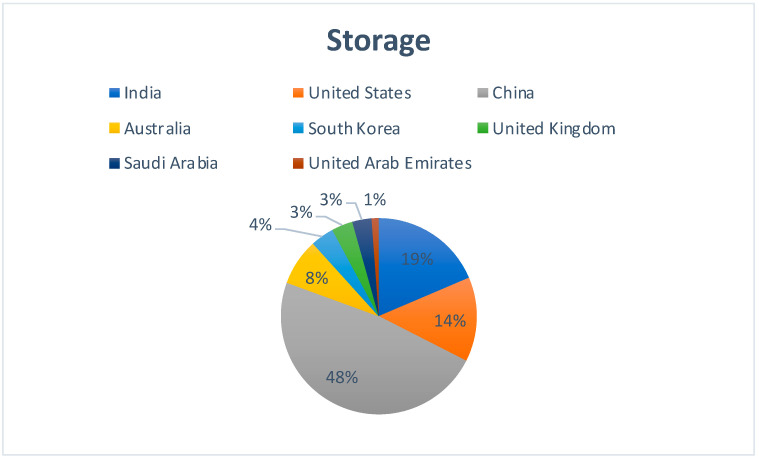
Region-specific publication distribution for the impact of Blockchain–Cloud integration on Cloud Storage.

Figure 13 represents the country-wise publication distribution for works concerning the applicability of Blockchain technology and Cloud storage. China has the highest contributions with 48% of the total publications.

### 8.4. Blockchain-as-a-Service

**Figure 14:**
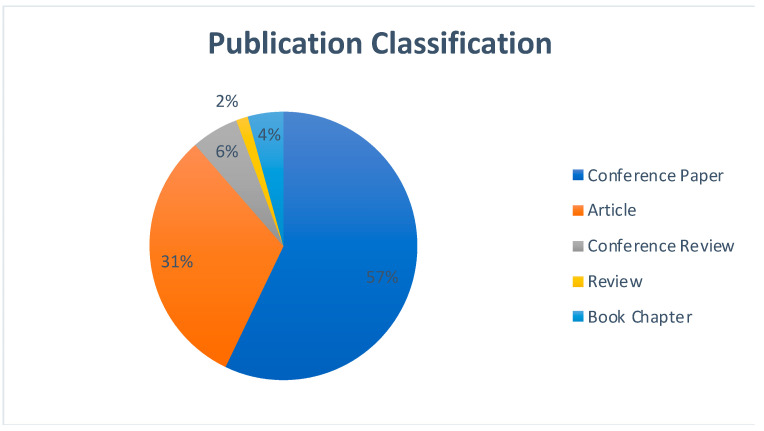
Publication classification comparison for Blockchain-as-a-Service.

Figure 14 represents the publication classification for the area of Blockchain-as-a-Service since the year 2017. The graph clearly indicates conference papers to be the largest section in terms of the works being published, followed by research articles. The graph suggests a rise in the number of conferences which are being conducted in the area of blockchain. Blockchain-as-a-Service being a recent research topic is attracting researchers to publish more of their work in the form of journal articles.

**Figure 15:**
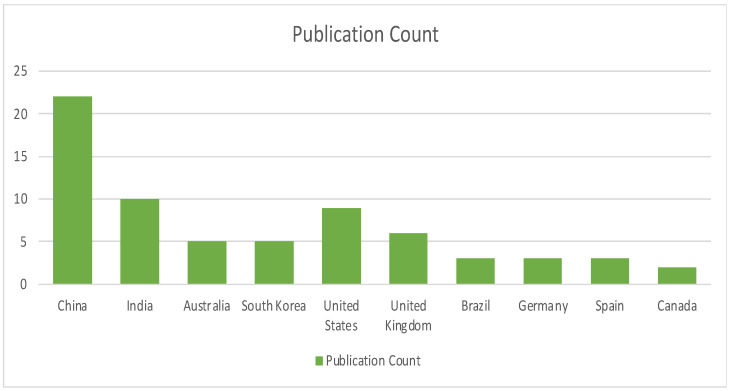
Region-specific publication distribution for Blockchain-as-a-Service.

Figure 15 illustrates the region-specific publication for Blockchain-as-a-Service in terms of publication count. Similar to other survey graphs, the trend continues to be in favor of China and India as the leading publication destinations.

### 8.5. Blockchain–Cloud Allied Technologies

**Figure 16:**
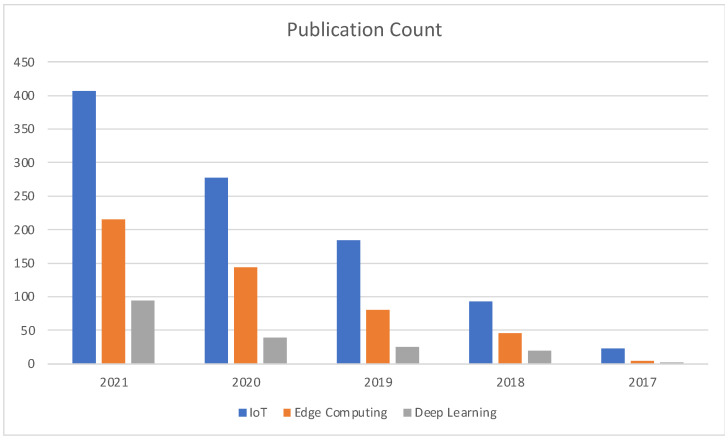
Year-specific Publication Count comparison among Allied Technologies.

Figure 16 illustrates the publication comparison between different allied technologies for Blockchain–Cloud integration. Our survey identifies the Internet of Things (IoT), Edge Computing, and AI/Deep Learning as the top three most prominent allied technologies that are currently being used in combination with Blockchain–Cloud integration.

**Figure 17:**
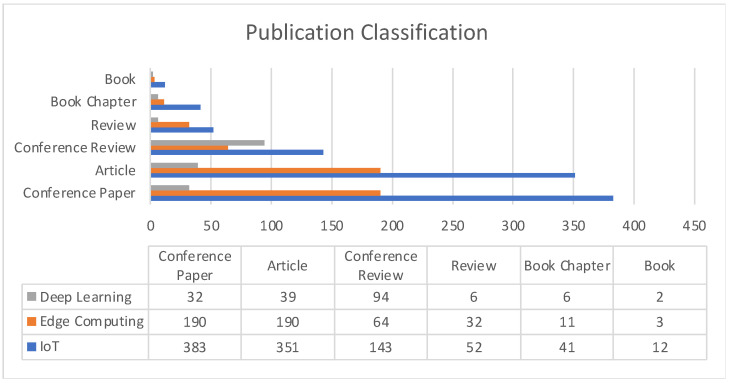
Publication classification comparison for Allied Technologies.

Figure 17 describes the publication distribution in terms of article classification such as conference papers, book chapters, articles, conference review papers, etc.

**Figure 18:**
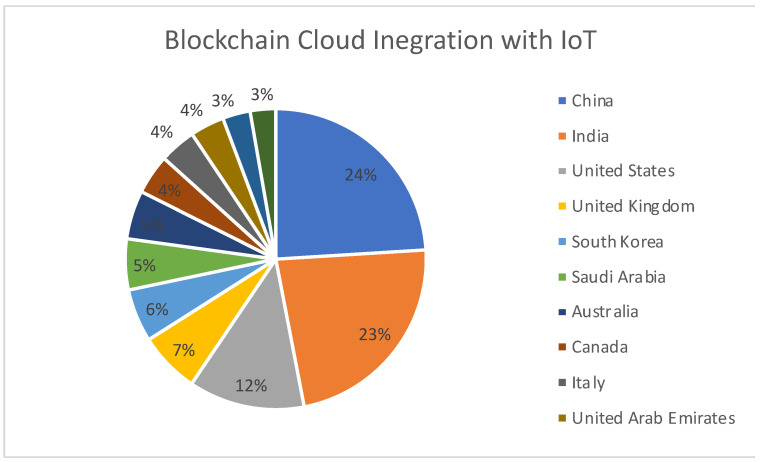
Region-specific publication distribution for Blockchain–Cloud integration with IoT.

Figure 18 represents the region-wise publication distribution for works concerning the use of Blockchain–Cloud integration along with the IoT. China and India are leading from the front, followed by the United States.

**Figure 19:**
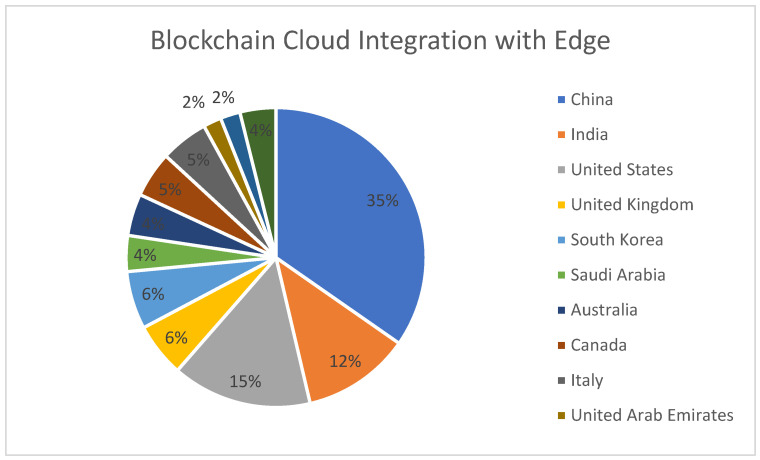
Region-specific publication distribution for Blockchain–Cloud integration with Edge Computing.

Figure 19 represents the region-wise publication distribution for works concerning the use of Blockchain–Cloud integration along with Edge computing. China is the most prominent region with the highest number of contributions.

**Figure 20:**
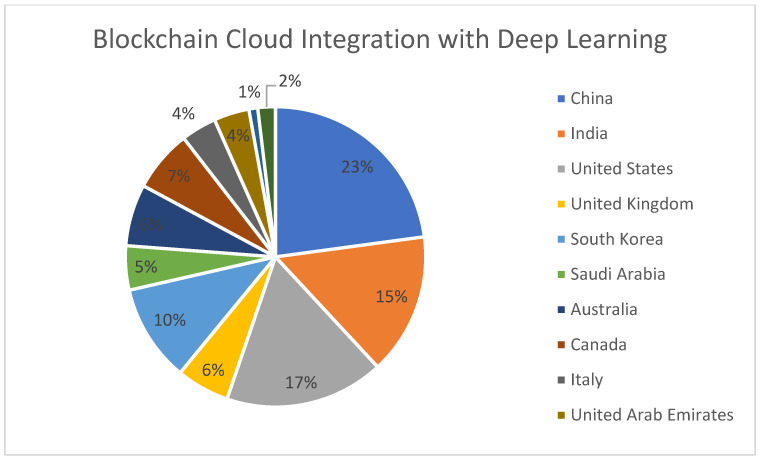
Region-specific publication distribution for Blockchain–Cloud integration with Deep Learning.

Figure 20 represents the region-wise publication distribution for works concerning the use of Blockchain–Cloud integration along with Deep Learning.

### 8.6. Research Growth Tajectorires

**Figure 21:**
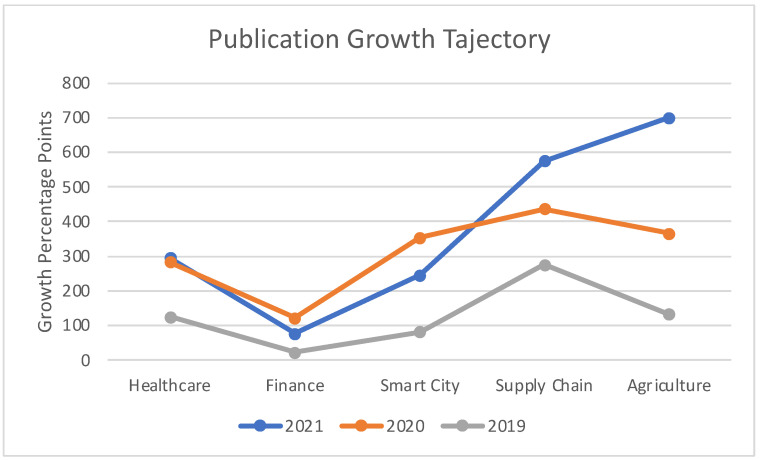
Comparison of Publication Growth Trajectory among Blockchain Application Areas.

Figure 21 illustrates a comparison between publication growth trajectories for different blockchain application areas. The figure represents a relative publication growth among application areas and aims to assist readers to pick and choose a specific application area for their research.

**Figure 22:**
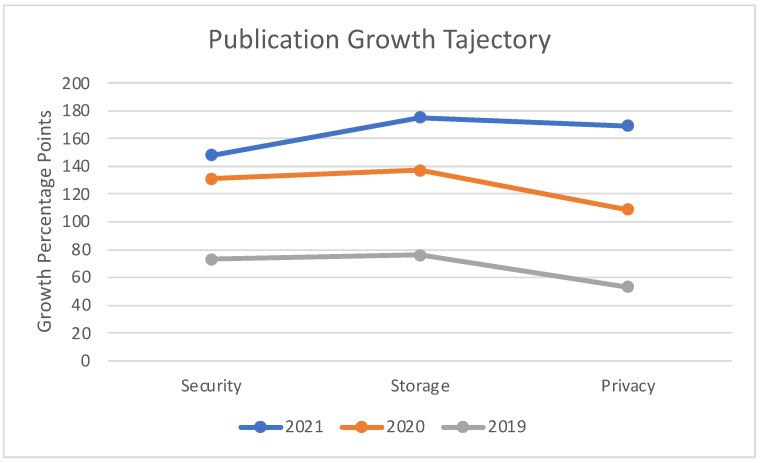
Comparison of Publication Growth Trajectory among Blockchain–Cloud Impact Areas.

Figure 22 illustrates a comparison between publication growth trajectories for different Blockchain–Cloud impact areas. The figure represents a relative publication growth among different Cloud impact areas and aims to assist readers to pick and choose a research problem in accordance with the publication growth trajectory of a specific impact area.

## 9. Industry Players and Services

**Figure 23:**
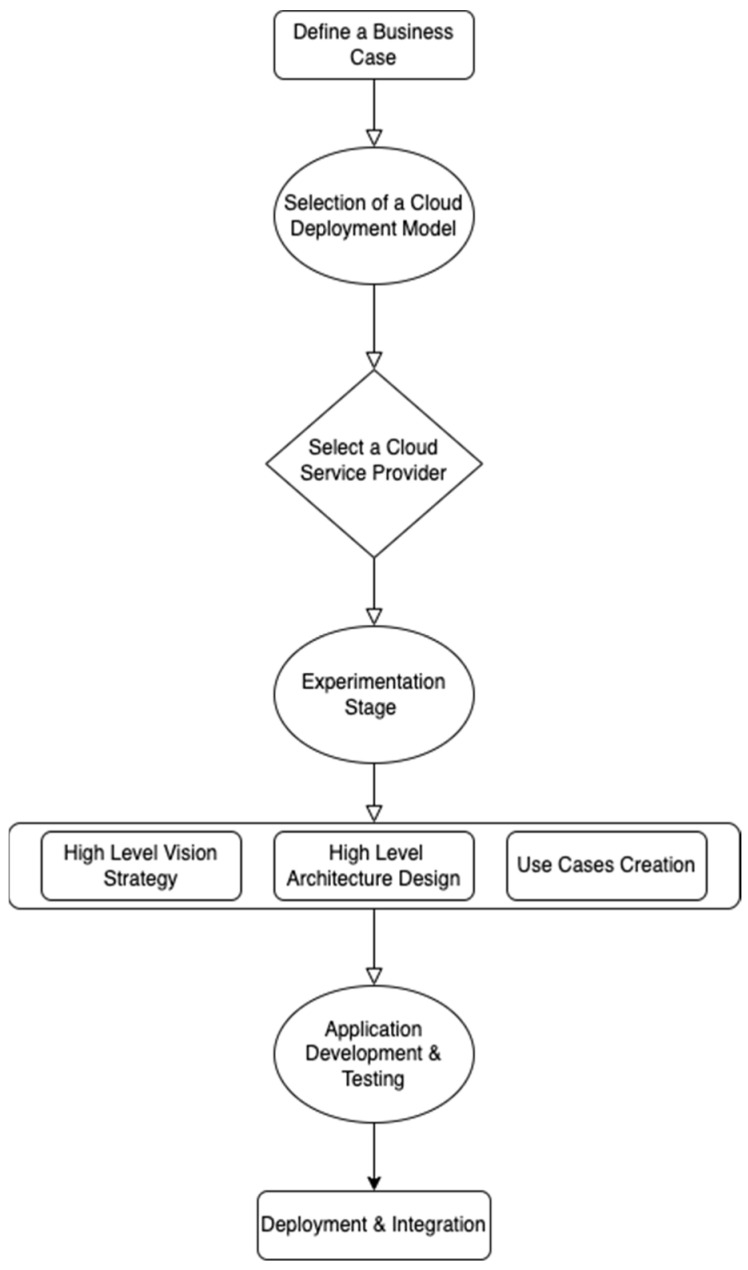
Blockchain–Cloud Adoption Journey.

The last couple of years have seen a widespread acceptance of blockchain across multiple domains, making it one of the fastest growing technologies in the IT industry. Seeing the rise of blockchain, many cloud service providers (CSP) came up with their own blockchain solutions popularly known as Blockchain-as-a-Service (BaaS) [101,102,103]. Many tech giants such as Microsoft, Google, Amazon, Oracle, and IBM have invested heavily in blockchain technology, which showcases their conviction towards the integration of Blockchain and Cloud. The CSP intend to provide enterprise grade solutions for blockchain coupled with the infrastructure support of Cloud. In this section, we discuss the three most prominent blockchain offerings by leading CSP and present a comparison among their blockchain solutions. Figure 23 illustrates the various stages of the Blockchain–Cloud adoption journey that any industry player can adopt or customize according to its own business requirements.

### 9.1. AWS Managed Blockchain

Amazon Web Services (AWS) launched its BaaS in the year 2018 in the form of Amazon Managed Blockchain. It is a fully managed service for creating and managing a blockchain network. AWS allows for the fast and scalable deployment of blockchain networks over EC2 (Elastic Compute Cloud) and EC3 instances. Amazon Managed Blockchain allows a user to choose between the two most popular blockchain frameworks, Ethereum and Hyperledger Fabric. The BaaS allows a developer to build distributed applications where multiple entities can securely perform transactions without the need for a central authority. The BaaS is offered as a pay-as-you-go service by Amazon, wherein the user only pays for the services being used and the amount of time they are being used for. AWS allows its users to customize their infrastructure in terms of variable CPU and memory configurations.

### 9.2. Azure Blockchain Service

Microsoft Azure blockchain was the pioneer cloud service provider to render BaaS for companies and developers. Azure promises its customers low-cost, fast, and safe blockchain implementations. Talking in terms of a market cap, Azure has the largest when it comes to Blockchain-as-a-Service. The Azure blockchain provides pre-configured infrastructure and network resources to its customers, thereby ensuring reduced deployment time. It also provides a plethora of internal tools for the fast and secure development of decentralized applications. The central cloud repository allows users to store their data in a secure manner. Azure Blockchain Service offers two kinds of services: Basic and Standard. The service tiers are divided based upon the performance and capabilities the services can offer. The basic services are meant for lightweight development and test workloads whereas the standard services can support the deployment of large production workloads.

### 9.3. Oracle Cloud Blockchain

Oracle came up with its blockchain solution soon after the launch of the Azure Blockchain Service. It named its blockchain service Oracle Blockchain Platform, which was an enterprise-grade solution focusing on the needs of enterprises and businesses. The service is a platform comprising of a distributed ledger that allows users to build and deploy blockchain applications over the cloud. The blockchain offering uses Hyperledger Fabric as the base framework for deploying blockchain networks. The Oracle Blockchain Platform allows its users to access numerous services of Oracle Cloud through in-house REST API gateways. Identity management, Oracle DB, and on-chain access control are some of the prominent built-in features of the blockchain platform. Table 4 illustrates a comparative analysis among key industry players rendering Blockchain-Cloud integration services.

## 10. Discussion and Conclusions

Blockchain is one of the fastest emerging technologies which has started to see its applicability beyond the financial sector. Its characteristics, such as immutability, data traceability, and security and its decentralized nature, have been the major driving factors for ensuring its success. Despite the merits of blockchain technology, there are a few challenges of scalability, energy consumption, and infrastructure requirements. It is believed that the integration of the Cloud with Blockchain can mitigate these challenges and enhance the development and deployment of decentralized applications. This work of ours aims to identify the extent of research that has been conducted concerning Blockchain–Cloud integration in the last few years. The paper presents case studies discussing Blockchain–Cloud integration that help to enhance the understanding of its readers. A detailed survey is conducted to examine the publishing patterns in the areas of Blockchain technology coupled with Cloud computing, Healthcare, Smart cities, and Finance. Furthermore, we discuss the concept of Blockchain-as-a-Service (BaaS) and explore key cloud service providers (CSP) which are offering blockchain services. The work presents a literature survey of publications concerning the implementation of BaaS along with a comparison between various blockchain services being offered by the CSP. The study performed presents us with a few questions to further investigate. What are the challenges of Blockchain–Cloud integration? What are the future application areas for BaaS? What kind of pricing and SLA policies can be created for ensuring efficiency and QoS? What type of architecture can be created for rendering BaaS? Through this work, readers can obtain an insight into the existing literature and will be able to craft their research journeys to answer some of the above questions.

Author Insights:

- Blockchain and Cloud computing are complementary technologies that work on the principles of distributed computing. Key challenge areas of Cloud, i.e., security, privacy, and the heterogenous storage of big data, can be easily mitigated by its integration with Blockchain technology.
- Resource allocation policies and internal Cloud operations can become more efficient, secure, and robust by Cloud’s integration with Blockchain.
- Blockchain-as-a-Service (BaaS) is expected to see a surge as more and more industry players are moving towards offering BaaS-related services.
- Existing Cloud services may experience an improvement in QoS when rendered along with blockchain integration.
- New integration areas for application and creation that prove beneficial for both Cloud and Blockchain users need to be identified by researchers.
- Supply chain management and smart agriculture continue to be the top runners for application areas of Blockchain–Cloud integration, as they represent the maximum spike in terms of publication growth trajectories.
- Cloud data privacy and Cloud storage are two specific research areas which show maximum spikes in terms of publication growth trajectories and thus can be beneficial choices as research problem areas.
- New models and frameworks are being proposed for the creation of smart cities involving the use of disruptive technologies such as Blockchain, IoT, AI, and Cloud computing.
- New Cloud governance models can be proposed by making use of blockchain consensus algorithms, thereby allowing multiple levels of customized data preferences, access control, and resource validation.
- IoT is the most prominent allied technology which has seen significant traction from researchers across the world when it comes to creating solutions in collaboration with Blockchain–Cloud integration.
- Researchers can work on problems involving the amalgamation of AI and Blockchain–Cloud integration, as it is the newest entrant to the club of allied technologies and has a significant research potential.
- Researchers can focus on devising efficient smart contracts that can facilitate the integration of both technologies by means of the efficient allocation of Cloud resources.
- Blockchain implementation on Hybrid Cloud models is an area for identifying new research possibilities.
- Scalability continues to be an open research challenge for Blockchain–Cloud systems in situations of varied heavy workloads.

## Figures and Tables

**Table 1:** Blockchain impact areas in Cloud Computing.

	Theme	Publications
Cloud Impact Areas	Security	[9,10,11,12]
	Privacy	[13,14,15,16,17]
	Storage	[18,19,20,21,22,23,24]

**Table 2:** Blockchain–Cloud Application Areas.

	Theme	Publications
Blockchain and Cloud	Healthcare	[34,35,36,37,38,39,40,41]
	Supply Chain	[42,43,44,45,46,47]
	Finance	[48,49,50,51]
	Smart Cities	[52,53,54,55,56,57,58,59]
	Agriculture	[60,61,62,63,64,65]

**Table 3:** Blockchain–Cloud Integration with Allied Technologies.

Serial No	Allied Technology	Cloud Deployment Model	Cloud Impact Area	Blockchain Type	Blockchain Platform
[85]	IoT, AI	Private Cloud	Computation	Private	Ethereum
[86]	IoT	Public/Private Cloud	Storage	Private	-
[87]	IoT, Deep Learning	Public Cloud	Security, Privacy	Private	Ethereum
[88]	IoT	Private Cloud	Security	Consortium	Ethereum
[89]	IoT	Private Cloud	Security, Storage	Consortium	-
[90]	Edge Computing	Public Cloud	Computation	Consortium	-
[91]	Edge Computing	Public Cloud	Computation	-	-
[92]	Edge Computing	Private Cloud	Security, Storage	Multichain	-
[93]	AI, Edge Computing	Private Cloud	Computation, Security, Privacy	Public, Private	-
[94]	AI, IoT	Public Cloud	Computation, Storage	Public	Customized Blockchain
[95]	Edge Computing, AI	Public Cloud	Privacy	Private	Ethereum
[96]	AI, IoT	Private Cloud	Security, Computation	Public	-
[97]	IoT	Public/Private Cloud	Security, Privacy, Storage	Public	-
[98]	Edge Computing, Deep Learning	Public Cloud	Security, Computation	Private	Ethereum
[99]	IoT, Edge Computing	Private Cloud	Security, Privacy	Private	-

**Table 4:** Comparative analysis of blockchain services by Cloud Providers.

Factor	AWS Blockchain	Azure Blockchain Service	Oracle Blockchain Platform
Blockchain Hosting Platform	Ethereum, Hyperledger Fabric, Corda	Ethereum, Hyperledger Fabric, Corda, MultiChain	Hyperledger Fabric
Blockchain Type	Permissioned	Permissioned	Permissioned, Private, Consortium
Key Services/Products	Amazon Quantum Ledger Database (QLDB), Amazon Managed Blockchain	Azure Blockchain Workbench, Ethereum on Azure	Blockchain App Builder, Blockchain Tables, Oracle PaaS
Architectural Design	Component based architecture	Layered Architecture	Layered Architecture
Pricing	Pay-as-you-go	Subscription plan, Pay-as-you-go	Pay-as-you-go, Subscription based, Licenced
Major Clients	Accenture, Nestle, GE Aviation, PHILIPS, SonyMusic	J.P. Morgan, Singapore Airlines, XBOX, Starbucks	iPoint, M2O, SERES, AJIB
